# Trends in energy and nutrient supply in Ethiopia: a perspective from FAO food balance sheets

**DOI:** 10.1186/s12937-019-0471-1

**Published:** 2019-08-13

**Authors:** Tony Sheehy, Emma Carey, Sangita Sharma, Sibhatu Biadgilign

**Affiliations:** 1 School of Food and Nutritional Sciences, University College Cork, Cork, Republic of Ireland; 2 Department of Medicine, Faculty of Medicine and Dentistry, University of Alberta, 5-10 University Terrace, 8303-112 St, Edmonton, AB T6G 2E1 Canada; 3 Friedman School of Nutrition Science and Policy, Tufts University, P.O. Box 24414, Addis Ababa, Ethiopia

**Keywords:** FAOSTAT, Food balance sheets, Nutrition transition, Ethiopia

## Abstract

**Background:**

Ethiopia is the second-most populous country in Africa. Although most people still live in rural areas, the urban population is increasing. Generally, urbanisation is associated with a nutrition transition and an increase in risk factors for non-communicable diseases (NCDs). The objective of this study was to determine how the nutritional composition of the Ethiopian food supply has changed over the last 50 years and whether there is evidence of a nutrition transition.

**Methods:**

Food balance sheets for Ethiopia from 1961 to 2011 were downloaded from the FAOSTAT database and daily per capita supply for 17 commodity groupings was calculated. After appropriate coding, per capita energy and nutrient supplies were determined.

**Results:**

Per capita energy supply was 1710 kcal/d in 1961, fell to 1403 kcal/d by 1973, and increased to 2111 kcal/d in 2011. Carbohydrate was by far the greatest energy source throughout the period, ranging from 72% of energy in 1968 to 79% in 1998; however, this was mostly provided by complex carbohydrates as the contribution of sugars to energy only varied between 4.7% in 1994 and 6.7% in 2011. Energy from fat was low, ranging from 14% of energy in 1970 to 10% in 1998. Energy from protein ranged from 14% in 1962 to 11% in 1994. Per capita supplies of calcium, vitamin A, C, D, folate and other B-vitamins were insufficient and there was a low supply of animal foods.

**Conclusions:**

The Ethiopian food supply is still remarkably high in complex carbohydrates and low in sugars, fat, protein, and micronutrients. There is little evidence yet of changes that are usually associated with a nutrition transition.

## Background

Over the last 50 years, dietary patterns around the world have changed dramatically [1–14]. Starting in developed countries, and more recently in developing countries, a pattern of “Westernisation” of the diet has emerged, with traditional, largely plant-based diets being replaced by increased intakes of animal products, fats and oils, highly processed foods (e.g. soft drinks, sweet or savoury snacks, reconstituted meat products, and pre-prepared frozen dishes), added sugars and salt, accompanied by a shift towards more sedentary work and leisure patterns. This phenomenon, known as the nutrition transition, generally occurs when a population moves from a predominantly rural, traditional lifestyle to an urban, industrial one [2, 14] and is nearly always preceded by epidemiological transitions in that population, such as declining fertility rates, lower maternal and infant mortality, reduced mortality from infectious diseases and increased life expectancy [15]. The major concern is that this transition is strongly associated with rising rates of obesity and other non-communicable chronic diseases (NCDs) [16]. By 2020, NCDs are expected to account for almost three-quarters of all deaths worldwide, and over 70% of deaths from ischaemic heart disease, stroke and type 2 diabetes will be occurring in developing countries [16]. Obesity – a major risk factor for NCDs - is already becoming a serious problem in parts of Africa despite the continued presence of undernutrition (defined as insufficient intake of energy and nutrients to meet an individual’s needs to maintain good health) [12, 17, 18], while all around the world the burden of obesity is shifting more towards the poor [11].

Ethiopia is the second-most populous country in Africa and the 13th most populous in the world, with an estimated population of 99.4 million in 2015 [19]. It is also one of the poorest countries in the world, with almost one-quarter of its people living on less than $1 a day [20]. The age profile of the population is very young (median age in 2015 was 18.3 years) and most people (> 80%) live in rural areas [21]; however, the urban population is increasing [20, 22]. Between 1990 and 2014 the urban population increased dramatically, from 6,064,000 (13% of total) to 18,363,000 (19%) and is forecast to reach 70,522,000 (39%) by 2050 [23]. Recent data show that 38% of children less than 5 years of age are stunted, 24% are underweight, 10% are wasted and more than 50% are anaemic, along with 18% of men and 23% of women in the 15–49-year age group [24]. Micronutrient deficiencies, including vitamin A, zinc, selenium, iron and iodine deficiency are major public health concerns [20, 25–29]. At the same time, risk factors for NCDs may be increasing, especially in urban areas. In 2005, 14% of urban women were overweight or obese compared with 2% in rural areas, and the highest prevalence (18%) was in the Addis Ababa region [30].

Because of the link between early childhood nutritional deprivation and later adult disease [31–33], the Ethiopian population, which is currently experiencing a high prevalence of fetal and post-natal growth retardation, will face an even greater risk for NCDs once these individuals reach adulthood. Thus, it is essential to monitor dietary trends in the country in order to identify the emergence of dietary patterns that are known to promote the development of NCDs. Dietary trends or food supply at a national level can be monitored crudely by the food disappearance method [34] using the food balance sheets that are produced annually by the United Nations Food and Agriculture Organisation (FAO) [35]. This method uses annual data on production and utilisation of all food commodities (including production within the country, imports, exports, stock changes, industrial non-food use, animal feed use, seed use, and waste) to derive a value for the average per capita supply of each commodity. By inputting these values into an appropriate food composition table, the average per capita supply of nutrients can then be calculated. Although they only show per capita supply of food commodities and not the actual dietary intakes of individuals, food balance sheets provide useful and timely information that can lead to a better understanding of current nutrition-related problems at the country level and assist in the development of more effective national public health nutrition policies [36–44]. Therefore, the objective of the present study was to analyse the FAO food balance sheets for Ethiopia from 1961 to 2011 to determine what changes have taken place in the energy and nutrient supply in the country over the last 50 years and to investigate whether there is evidence of a nutrition transition.

## Methods

FAO food balance sheets for Ethiopia from the period of 1961–2011 were downloaded from the FAOSTAT database [45]. Up to and including 1992, the Ethiopian food balance sheets include data from Eritrea, but not thereafter, as Eritrea gained independence from Ethiopia in 1993. These food balance sheets provide the overall per capita supply (as kilogram (kg)/year) for 98 food commodities, including cereals, starchy roots, vegetables, fruits, oilseeds and oilseed products, tree nuts, animal fats, milk, meats, eggs, and fish. The full list of commodities is shown in Appendix 1. After importing the data into Microsoft® Office Excel 2010 (Microsoft Corp., Redmond, WA, USA), we converted per capita supply (kg/year) to grams/day (g/d).

To evaluate the trends in energy and nutrient supply we constructed a food composition table in Microsoft® Office Excel 2010 and matched the commodities on the food balance sheets with appropriate foods from McCance and Widdowson’s The Composition of Foods, 5th and 6th editions plus supplements [46], or, in a small number of cases, the USDA food composition tables [47]. For consistency, foods were coded as being in their least processed form. For example, sweet potatoes were coded as ‘sweet potatoes, raw’, barley as ‘barley, whole grain, raw’, and eggs as ‘eggs, chicken, whole, raw’. Certain broad categories of commodity in the food balance sheets, such as peas, beans, nuts, fish, and those labelled ‘other’, are lacking in detail about the specific foods that make up the category. To code these categories in a way that would be region-specific, we reviewed food lists of food frequency questionnaires and other relevant literature from Ethiopia and neighbouring countries [20, 48–52] in order to identify commonly consumed foods that belonged in that category. For categories where this procedure was carried out, supply was divided equally among the constituent items. In total, 53 commodities were matched with foods from McCance and Widdowson’s Food Composition Tables, 4 commodities (sorghum and products, cottonseed, fish body oil and ricebran oil) were matched with foods from the USDA food composition database, and 27 commodities were matched to composite codes that we created. The remaining 14 commodities, including such items as alcohol (non-food), sugar beet, sugar cane, aquatic plants, aquatic mammals (other), and infant food, were not coded because they contributed little or nothing to the Ethiopian food supply: for most of these commodities no values at all were provided by the food balance sheets for the entire period under investigation, while for the remainder, supply was either zero or else a very small supply (≤2.5 kg/capita/year) was recorded during certain specific years. Depending on the year, the commodities we coded accounted for between 97 and 99.3% of the total energy supply.

### Statistical analysis

The FAO food balance sheets themselves provide estimates of per capita energy, protein and fat supply; however, they do not provide any figures for carbohydrate supply. In order to check the level of agreement between the FAO estimates for energy, protein and fat and our calculated values we obtained Pearson correlations using Microsoft® Office Excel 2010. Statistical significance of correlations was accepted at the 5% level. All tests were two-sided.

## Results

### Correlations between our calculated values for energy, fat and protein supply and the FAO estimates

**Fig. 1:**
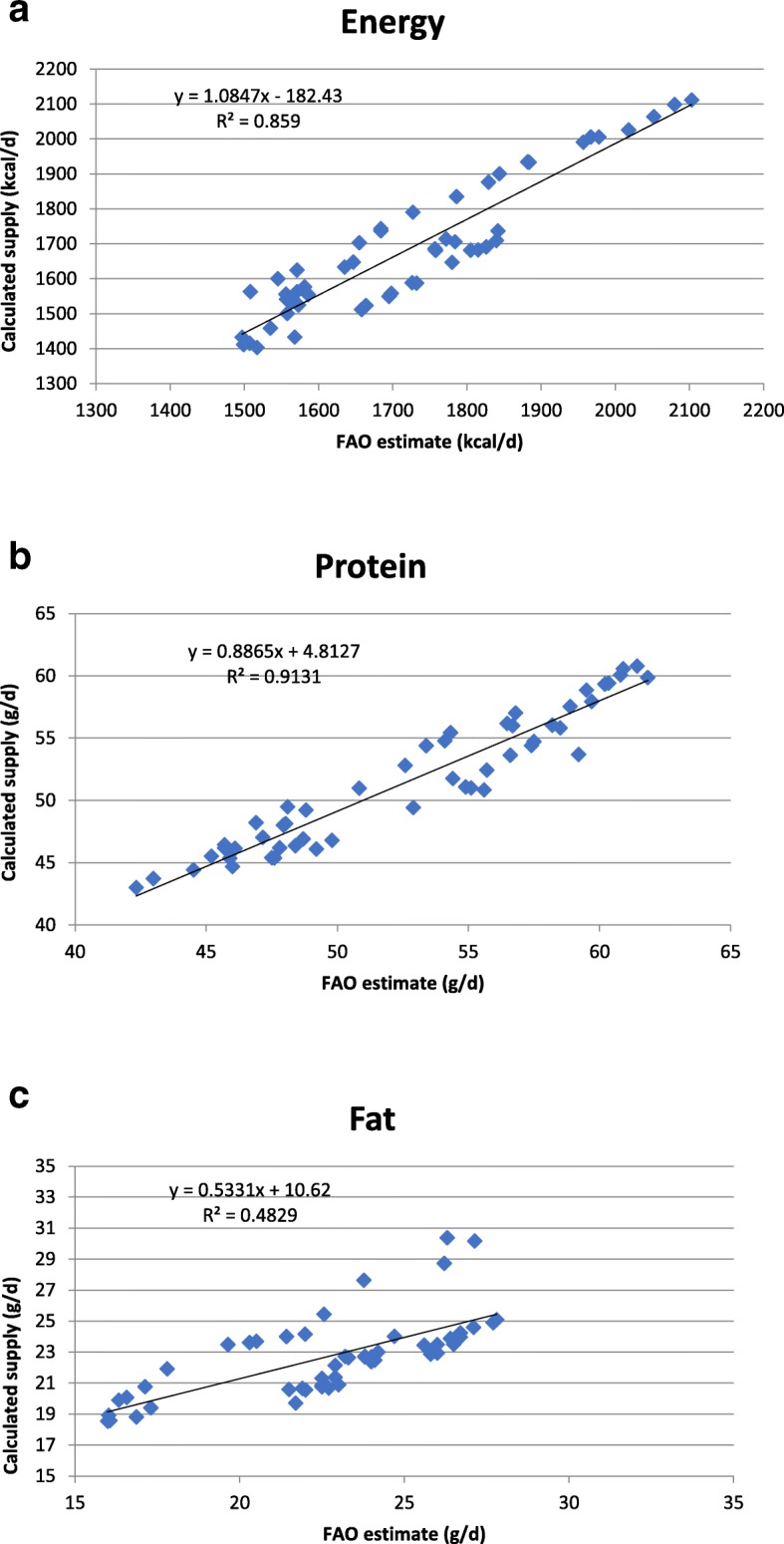
**a**: Relationship between per capita energy supply shown on the FAO food balance sheets and our calculated values. **b**: Relationship between per capita protein supply shown on the FAO food balance sheets and our calculated values. **c**: Relationship between per capita fat supply shown on the FAO food balance sheets and our calculated values

Figure 1(a) shows the correlation between the per capita energy supply as reported on the FAO food balance sheets and the values we calculated using the food composition database we constructed specifically for this study. On average, our values for energy were 2.1% lower than the food balance sheet estimates, but the correlation between them was statistically significant (r = 0.927, *P* = 0.0000). Our values for fat supply were, on average, 0.1% higher and our values for protein were 2.2% lower than the food balance sheet estimates; however, again, there was a strong and significant correlation between them (*r* = 0.695, *P* = 0.0000 for fat and r = 0.956, *P* = 0.0000 for protein) (Fig. 1b and c).

### Trends in energy and macronutrient supply

**Fig. 2:**
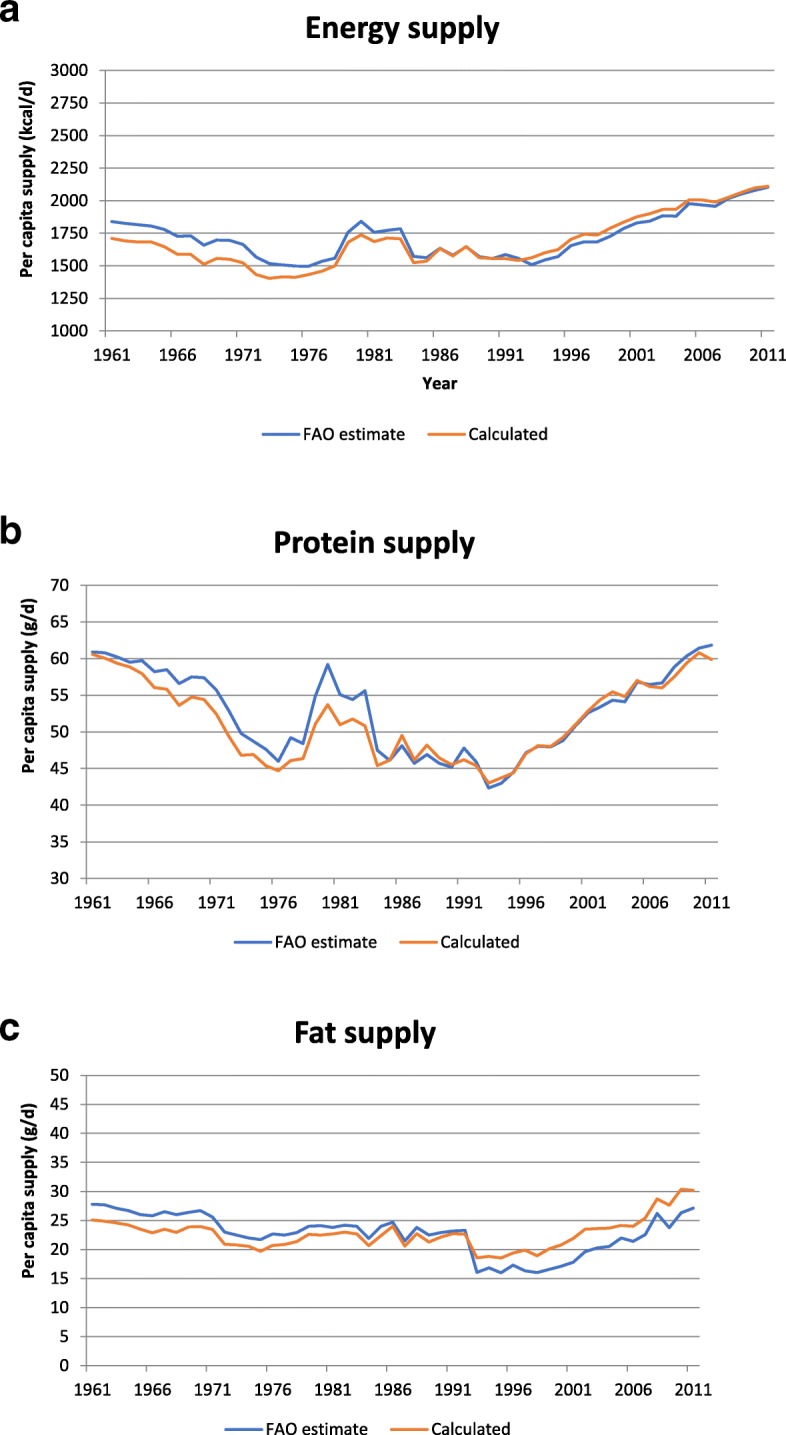
**a**: Food balance sheet-derived and our calculated values for per capita energy supply in Ethiopia between 1961 and 2011. **b**: Food balance sheet-derived and our calculated values for per capita protein supply in Ethiopia between 1961 and 2011. **c**: Food balance sheet-derived and our calculated values for per capita fat supply in Ethiopia between 1961 and 2011

The 50-year trends in per capita energy supply in Ethiopia between 1961 and 2011 are shown in Fig. 2(a). According to our calculations, per capita energy supply was 1710 kcal/d in 1961 and it fell to as low as 1403 kcal/d by 1973. Since then there has been an increase in per capita energy supply, especially since the early 1990s, so that by 2011 the value was about 50% higher at 2111 kcal/d. The trends according to the FAO food balance sheets estimates are also shown in Fig. 2(a) for comparison. Our values were slightly lower than the FAO values until the mid-1970s, but there was excellent agreement between the two datasets after that.

The 50-year trends in per capita supply of protein and fat in Ethiopia are shown in Fig. 2(b) and (c), respectively. According to our calculations, between 1961 and 1976, per capita protein supply fell from 61 g/d to 45 g/d, and per capita fat supply fell from 25 g/d to about 20 g/d. From 1976 to the early 1990s there were fluctuations in the supply but little evidence of any major reversal in the trend. However, since 1993 the per capita supplies of both protein and fat have been increasing, reaching 60 g protein and 30 g fat/d by 2011. Carbohydrate supply also fell during the 1960s and early 1970s, from 327 g/d in 1961 to 270 g/d in 1973 (data not shown); however, since then, carbohydrate supply has been on the rise and by 2011 it was more than 50% higher, at 421 g/d. The FAO food balance sheets do not provide estimates of carbohydrate supply but their estimates for fat and protein are shown in Fig. 2(b) and (c); both sets of figures are in good agreement with our calculated values.

### Contributions of macronutrients to energy supply

**Fig. 3:**
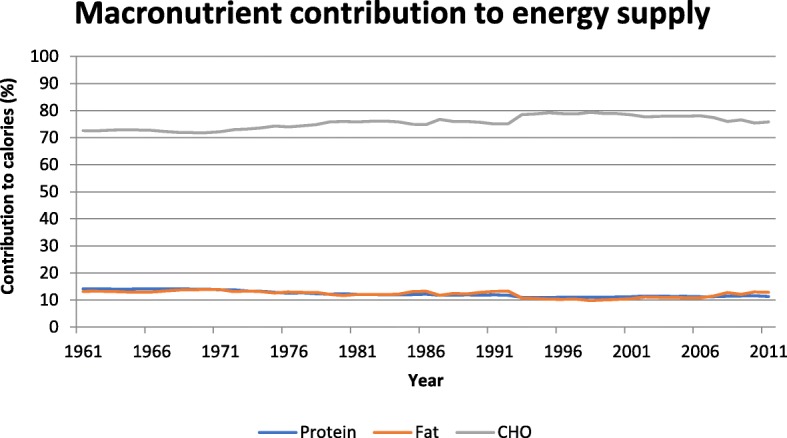
Contributions of protein, fat and carbohydrate to energy supply (%) in Ethiopia between 1961 and 2011

The contribution of proteins, fats and carbohydrates to total energy supply in Ethiopia from 1961 to 2011 is shown in Fig. 3. Carbohydrate was by far the biggest energy source throughout the period, ranging from 72% of energy in 1968 to 79% in 1998. Energy from fat was exceptionally low, ranging from 14% of energy in 1970 to only 10% in 1998. Energy from protein ranged from 14% in 1962 to 11% in 1994. Alcohol made a very minor contribution to energy, ranging from < 0.2% in 1995 to a maximum of about 0.7% in 1976 (data not shown).

### Trends in fatty acid supply and P:S ratio

**Fig. 4:**
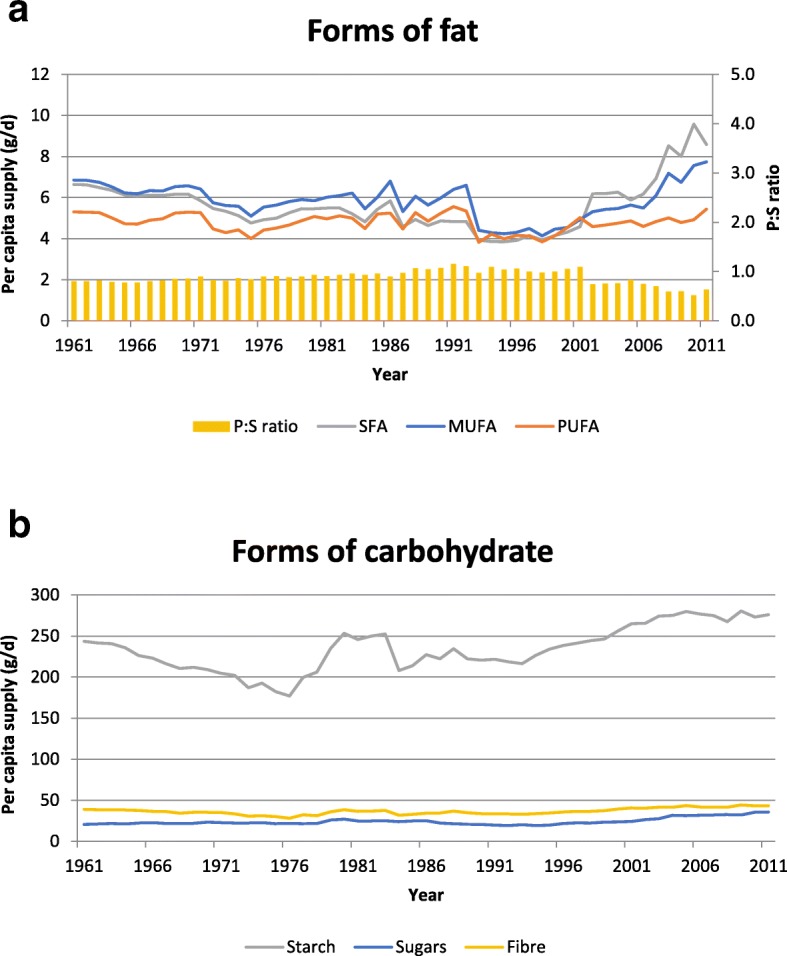
**a**: Per capita supply of SFA, MUFA and PUFA (g/d) and polyunsaturated-to-saturated (P:S) ratio in Ethiopia between 1961 and 2011. **b**: Per capita supply of sugars, starch and fibre (g/d) in Ethiopia between 1961 and 2011

The 50-year trends in saturated (SFA), monounsaturated (MUFA) and polyunsaturated (PUFA) fatty acid supply in Ethiopia and in the polyunsaturated-to-saturated (P:S) ratio are shown in Fig. 4(a). In general, per capita fatty acid supply - which was already low - declined between 1961 and the mid-1990s and there was a slight increase in the P:S ratio, from a minimum of 0.77 to a maximum of 1.15. These trends have now reversed and since 1998 there has been a consistent, albeit small, increase in fatty acid supply and a fall in the P:S ratio.

### Trends in starch, sugar and fibre supply

The 50-year trends in starch, sugars and fibre supply in Ethiopia between 1961 and 2011 are shown in Fig. 4(b). Starch was the major form of carbohydrate while sugars made up less than 10% of the total carbohydrate supply throughout the period. There was a fall in per capita starch supply from 243 g/d in 1961 to 177 g/d in 1976, followed by a sharp increase to 253 g/d in 1980 and a more gradual increase over recent decades, reaching 276 g/d in 2011. The maximum value for per capita fibre supply (44 g/d) was recorded in 2009 and the minimum value (28 g/d) was in 1976. Per capita fibre supply in 2011 was 43 g/d.

### Trends in micronutrient supply

**Fig. 5:**
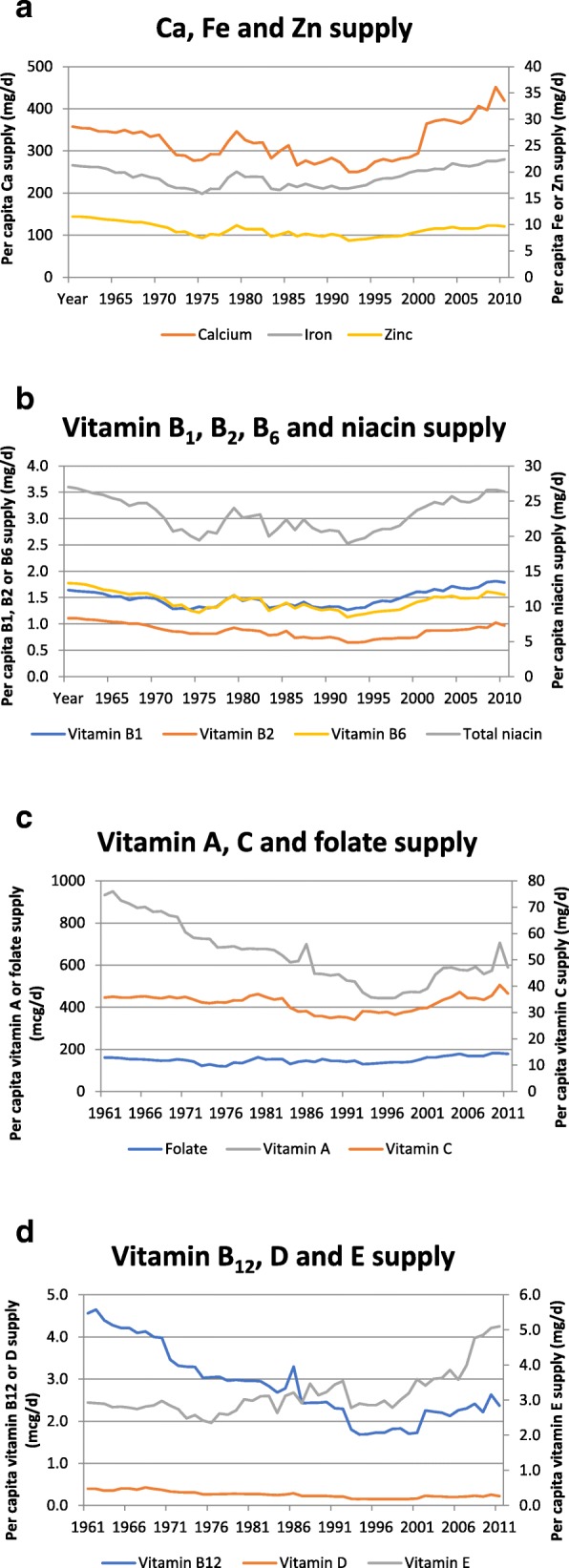
**a**: Per capita supply of calcium, iron and zinc (mg/d) in Ethiopia between 1961 and 2011. **b**: Per capita supply of vitamin B_1_, B_2_, B_6_ and niacin (mg/d) in Ethiopia between 1961 and 2011. **c**: Per capita supply of vitamin A (mcg/d), C (mg/d) and folate (mcg/d) in Ethiopia between 1961 and 2011. **d**: Per capita supply of vitamin B_12_ (mcg/d), D (mcg/d) and E (mg/d) in Ethiopia between 1961 and 2011

The 50-year trends in micronutrient supply in Ethiopia between 1961 and 2011 are shown in Fig. 5(a)-(d). Calcium (Ca), iron (Fe), zinc (Zn), vitamin A, B_1_, B_2_, B_6_, B_12_, niacin, folate, C and D supplies were already low in the 1960s and all declined during the next 2 decades before eventually stabilising and starting to increase from the mid-1990s onwards. The trend for vitamin E was slightly different, as minimum supply occurred earlier, in the mid-1970s.

### Trends in supply of major commodities

**Fig. 6:**
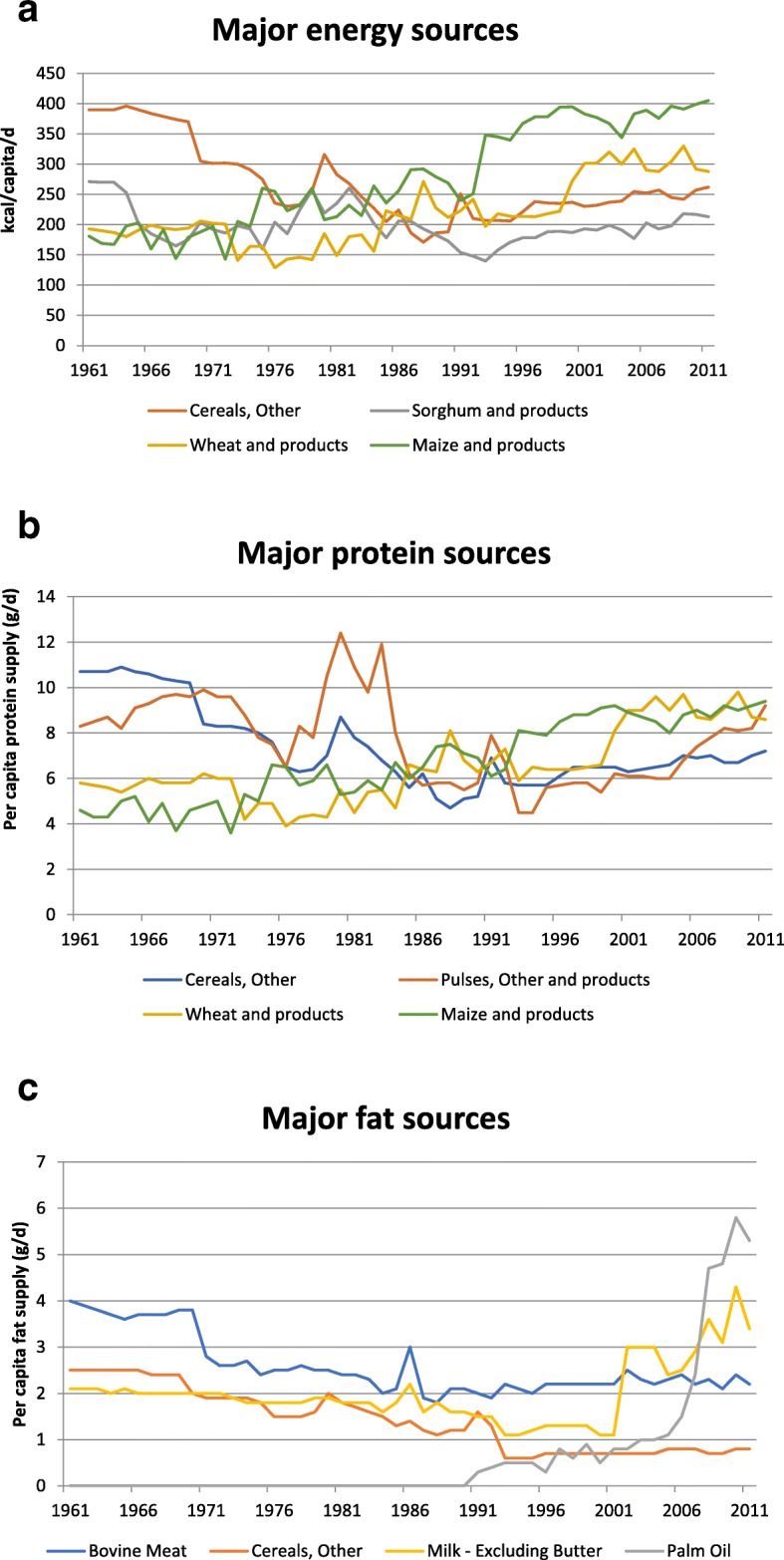
**a**: Major sources of energy (kcal per capita/d) in Ethiopia between 1961 and 2011. **b**: Major sources of protein (g per capita/d) in Ethiopia between 1961 and 2011. **c**: Major sources of fat (g per capita/d) in Ethiopia between 1961 and 2011

The commodities providing the most energy in the Ethiopian food supply between 1961 and 2011 are shown in Fig. 6(a). Cereals have been, and remain, by far the major contributors to energy although there has been a change in their relative contributions over time. During the 1960s and 1970s ‘Cereals, other’ (which in the Ethiopian context is predominantly teff) was the major energy source, but since the 1980s this has been overtaken by wheat and maize. Sorghum was the second most important contributor to energy supply in the early 1960s, but its importance has diminished over time, although it still ranks fourth in terms of calories provided. Cereals (along with pulses) are also the major sources of protein in Ethiopia, and even in 2011 there was no animal food among the top 5 protein sources (Fig. 6(b)). In the early 1960s, bovine meat was the major source of fat but in recent decades it has been overtaken by palm oil and milk (Fig. 6(c)).

## Discussion

The objective of the present study was to analyse the FAO food balance sheets for Ethiopia from 1961 to 2011 in order to determine what changes have taken place in the country’s dietary energy and nutrient supply over the past 50 years. Our expectation was that if a nutrition transition is occurring this should be evident as an increase in the overall supply of energy and in the percentage of energy coming from fat and sugars, a fall in the percentage of energy coming from starch, and an increase in the usage of vegetable oils and animal-derived foods. Our analysis revealed that although the per capita energy supply in Ethiopia has increased substantially over the course of the last two decades, cereals remain the major contributors to dietary energy and indeed protein, and there has only been a small increase in energy from sugars and in the usage of vegetable oils and animal-derived foods. This suggests that although rapid urbanisation of the population is occurring, the country as a whole is still in the early stages of a nutrition transition.

On average, our calculated values for protein and energy supply were 2.2 and 2.1% lower, and our values for fat were 0.1% higher than those given on the food balance sheets. Both sets of values were significantly correlated and trends observed over time were virtually identical (Fig. 2(a)-(c)). This suggests that the way we coded the commodities was appropriate. The small differences in absolute values may be due to differences between the food composition tables upon which our analysis was based [46, 47] and the older nutrient values used in the FAO statistical databases over time. FAO cautions that for a variety of reasons these older compositional data may not be reflective, in many cases, of the foods and nutrients consumed today [53].

Our analysis showed that energy, protein and fat supply, which were already low, declined from the early 1960s until the early to mid-1970s (Fig. 2(a)-(c)). Since then, there has been an improvement in the situation, with an increase in energy supply of 570 kcal/capita/d or 40% being recorded since 1992–1993. This is consistent with trends internationally, as Kennedy [54] reported that the global per capita energy supply increased by some 500 kcal/d between 1961 and 1999. Also, results from the Ethiopian Household Income Expenditure Surveys showed an increase of almost 700 kcal/adult equivalent between 1995 and 96 and 2004–05 [55]. Protein supply in Ethiopia has also increased by 40% since the early 1990s but despite this, the prevalence of undernourished people in Ethiopia is still reported to be one of the highest in East Africa [56]. (FAO uses the Prevalence of Undernourishment indicator to estimate the extent of chronic hunger in the world, thus “hunger” - i.e. insufficient consumption of dietary energy - may also be referred to as undernourishment.)

The WHO recommends that 55–70% of dietary energy should come from carbohydrate (with < 10% coming from free sugars, which it defines as “all monosaccharides and disaccharides added to foods by the manufacturer, cook or consumer, plus sugars naturally present in honey, syrups and fruit juices”) and 15–30% should come from fat [16]. Our data show that unlike most countries, which are experiencing increasing fat and decreasing carbohydrate supply, the food supply in Ethiopia has, throughout the last 50 years, exceeded the WHO recommendation for energy from carbohydrate and fallen below their recommendation for energy from fat. Within the last decade and a half, starting from the mid-1990s, energy from carbohydrate has been falling slowly and energy from fat has been increasing (notably from palm oil), and there has been a downward shift in the P:S ratio to less than 1.0. This may indicate the very early stages of a nutrition transition similar to what has been experienced elsewhere, but the food supply is still remarkably high in starch (from cereals and starchy roots) and low in fat, protein and sugars. For example, our data show that in 2011 carbohydrates provided 75.8% of energy, while only 12.9% came from fat and 11.3% came from protein; moreover, sugars provided only 6.7% of energy. Using different methodology, broadly similar findings were reported by the Ethiopia National Food Consumption Survey [57]. Based on a single 24-h recall collected between June and September 2011 in a nationally representative sample of 6702 women of childbearing age, the contributions of carbohydrates, fats and proteins to energy were 73.5, 16.5, and 9.7% respectively. Interestingly, however, in their smaller sample (*n* = 377) of urban men, carbohydrates provided only 68.1% of energy, while the contribution from fat increased to 20.7%.

The WHO recommendation for fibre intake is > 25 g/d of total dietary fibre [16]. Our data show that per capita supply of fibre has been increasing since the mid-1970s and was about 43 g/d in 2011; however, it is important to recognise that food balance sheets do not take into account the losses that occur beyond the retail level such as those due to peeling, food preparation and wastage, so this figure is likely to be an over-estimate of true dietary intake.

The present results (Fig. 5(a)–(d)) show that per capita supply for a number of important micronutrients increased slowly over the last two decades, in line with the general increase in energy supply. However, supplies of calcium, vitamin B_2_, folate and vitamin C are still below the WHO and FAO nutrient intake recommendations [58] (Appendix 2) - substantially in the case of calcium and folate - while vitamin A and B_6_ are borderline. Given the low supply of animal foods and the low bioavailability of iron and zinc from plant-based diets, these (along with vitamin B_12_ and D) are also nutrients of concern. Consistent with these results, a recent study on the micronutrient intakes of urban adults in Northern Ethiopia [27] reported inadequate intakes of calcium, retinol, vitamin B_1_, B_2_, niacin and vitamin C in the vast majority of study participants (73–100%, depending on the nutrient). The Ethiopia National Food Consumption Survey [57] reported a very high prevalence of inadequate intakes of vitamin A in women of childbearing age (81.9%) and in urban male adults (91.3%); moreover, zinc intakes were inadequate in 50.4% of women of childbearing age and in over 60% of urban adult males. The low supply of micronutrients reflects the fact that the Ethiopian diet continues to be composed mainly of cereals, roots, tubers, and pulses. There is low dietary diversity and low consumption of fruit and vegetables, fish and animal products, all of which are important sources of micronutrients.

This study does have limitations. Food balance sheets overestimate actual food consumption and nutrient intakes because they fail to take into account losses that occur beyond the retail level, such as wastage during food preparation, losses due to processing, food that is spoiled or simply not eaten, and food fed to animals in the home [39]. Also, no account is taken of regional differences in food supply within a country, or of differences between different age groups, social classes or rural versus urban dwellers. Thus, it is not possible to compare food balance sheet data directly with data from national food consumption surveys because each approach measures different levels of dietary information [59]. This challenge can be overcome to some extent by expressing results on an energy density basis, as % of total energy, or as ratios. A further limitation is the fact that the food balance sheets show basic commodities rather than specific food products, which poses a challenge when it comes to choosing the most appropriate codes for nutritional analysis. For consistency purposes, our approach was to code at the level of the raw unprocessed commodity wherever possible; however, this could result in an overestimation of some dietary components, such as fibre. The lack of detail regarding the specific foods that make up certain categories of commodity in the food balance sheets is another limitation. As in previous studies [44, 60, 61], we tried to overcome this limitation by populating these categories using information about commonly consumed foods from region-specific food frequency questionnaires and other relevant literature. Another potential limitation is the fact that the food composition table we constructed was based on UK rather than African food composition data; however, we would not expect the composition of basic commodities to differ all that much between countries, and the UK food composition tables are much more comprehensive than existing African food composition tables [62], which facilitates more accurate coding.

## Conclusions

In conclusion, unlike many lower- and middle-income countries which have experienced major shifts in the composition of their food supply over the last 50 years, the Ethiopian food supply is still remarkably high in complex carbohydrates (mainly from cereals, roots and tubers) and low in fat, protein and sugars. Since the early 1990s there has been an increase in the overall energy and protein supply, and the micronutrient supply has also improved, but it is still insufficient for calcium, vitamin A, folate and other B-vitamins. Iron and zinc bioavailability will continue to be compromised by the continuing high reliance on grains and pulses and low use of animal foods. The increased usage of maize and wheat at the expense of teff and the appearance of palm oil and milk as fat sources in recent years may be signalling the emergence of a more highly processed food supply, but there is little evidence yet of the kinds of changes that are usually associated with the nutrition transition. These data should provide a useful starting point for further and more detailed studies on diet and chronic disease associations in Ethiopia, and for developing nutrition and health promotion strategies. Owing to the inherent limitations of food balance sheets, further research should be carried out using different methodologies to corroborate these findings.

## Data Availability

Data will be available from the corresponding author upon request.

## Appendix 1

Commodities listed in FAO Food Balance Sheets.

CEREALS – EXCLUDING BEER: wheat; rice (milled equivalent); barley; maize; rye; cereals (other).

STARCHY ROOTS: cassava; potatoes; sweet potatoes; yams; roots (other).

SUGAR CROPS: sugar (raw equivalent); sweeteners (other); honey.

PULSES: beans; peas; pulses (other).

TREE NUTS.

OILCROPS: soyabeans; groundnuts (shelled equivalent); sunflowerseed; rape and mustardseed; coconuts - including copra; sesameseed; olives;

VEGETABLE OILS: soyabean oil; groundnut oil; sunflowerseed oil; cottonseed oil; palmkernal oil; coconut oil; sesameseed oil; olive oil; maize germ oil;

VEGETABLES: tomatoes; vegetables (other).

FRUITS – EXCLUDING WINE: oranges; lemons; grapefruit; citrus (other); bananas; plantains; apples; pineapples; dates; grapes; fruits (other).

STIMULANTS: coffee; cocoa beans; tea.

SPICES: pepper; pimento; cloves; spices (other).

ALCOHOLIC BEVERAGES: wine; beer; beverages (fermented); beverages (alcoholic).

MEAT: bovine meat; mutton & goat meat; pigmeat; poultry meat; meat (other).

ANIMAL FATS: butter, ghee; cream; animal fats (raw); fish body oil; fish liver oil;

MILK - excluding butter.

EGGS.

FISH, SEAFOOD: freshwater fish: demersal fish; pelagic fish; marine fish; crustaceans; cephalopods; molluscs; other.

## Appendix 2

**Table 1:** Recommended nutrient intakes (WHO & FAO, 2004)

Nutrient	Female (age 19–50 years)	Male (age 19–50 years)	Mean
Ca (mg)	1000	1000	1000
Fe (mg) – assuming;			
15% bioavailability	19·6	9·1	14·4
10% bioavailability	29.4	13.7	21.6
5% bioavailability	58.8	27.4	43.1
Zn (mg) – assuming;			
high bioavailability	3.0	4.2	3.6
mod. Bioavailability	4·9	7	6.0
low bioavailability	9.8	14.0	11.9
Vitamin A (μg retinol equivalents)	500	600	550
Vitamin B_1_ (mg)	1.1	1.2	1.15
Vitamin B_2_ (mg)	1.1	1.3	1.2
Vitamin B_6_ (mg)	1.3	1.3	1.3
Niacin (mg niacin equivalents)	14	16	15
Folate (μg dietary folate equivalents)	400	400	400
Vitamin C (mg)	45	45	45

## Abbreviations

Ca: Calcium
CSA: Central Statistics Agency
FAO: Food and Agriculture Organisation of the United Nations
Fe: Iron
MUFA: Monounsaturated fatty acid
NCD: Non-communicable diseases
PUFA: Polyunsaturated fatty acid
SFA: Saturated fatty acid
WHO: World Health Organisation
Zn: Zinc
